# AAV Gene Augmentation Therapy Rescues the Phenotype in the *RDH5*^−/−^ Cat Model of Fundus Albipunctatus

**DOI:** 10.1167/iovs.67.12.45

**Published:** 2026-10-21

**Authors:** Laura M. Ford, Kelian Sun, Laurence M. Occelli, Mary E. Enfield, Anahita Daruwalla, Ryan F. Boyd, Philip D. Kiser, Simon M. Petersen-Jones

**Affiliations:** 1 Genetics and Genome Sciences, Michigan State University, East Lansing, Michigan, United States; 2 Small Animal Clinical Sciences, Michigan State University, East Lansing, Michigan, United States; 3 Department of Physiology and Biophysics, University of California, Irvine, Irvine, California, United States; 4 Brunson Center for Translational Vision Research, Department of Ophthalmology and Visual Sciences, University of California, Irvine, Irvine, California, United States; 5 Ophthalmology Services, Charles River Laboratories, Mattawan, Michigan, United States; 6 Department of Clinical Pharmacy Practice, School of Pharmacy and Pharmaceutical Sciences, University of California, Irvine, Irvine, California, United States; 7 Research Service, VA Long Beach Healthcare System, Long Beach, California, United States

**Keywords:** *RDH5*, fundus albipunctatus, AAV2, gene therapy, large-animal cat model

## Abstract

**Purpose:**

Retinol dehydrogenase 5 (RDH5) catalyzes the final oxidation for visual pigment regeneration of 11-*cis*-retinal as part of the visual cycle in the retinal pigment epithelium (RPE). In humans, *RDH5* mutations cause fundus albipunctatus characterized by night blindness, accumulation of white-yellowish fundus spots, and, in a subset of patients, macular degeneration. Currently, there is an unmet need for therapies for patients with *RDH5* mutations. Using a large-animal cat model of *RDH5*-associated disease (*RDH5*^−/−^), this study aimed to meet these needs through development of a gene augmentation therapy able to rescue *RDH5*-associated retinopathies.

**Methods:**

An adeno-associated virus serotype 2 with triple capsid mutations vector packaged with feline *RDH5* cDNA (AAV-*VMD2*-*fRDH5*) was subretinally injected into 12 *RDH5*^−/−^ cats. To assess expression and the impact of treatment on retinal function and structure, immunohistochemistry, high-performance liquid chromatography, rod and cone electroretinograms, spectral-domain optical coherence tomography, and semi-thin resin-embedded sectioning were performed.

**Results:**

The expression of the *RDH5* transgene improved rod and cone photoreceptor functional recovery following light exposure and shows promise in slowing/halting area centralis (analogous to the human macula) degeneration.

**Conclusions:**

This gene augmentation therapy is a promising approach for the treatment of *RDH5*-associated retinopathies.

Retinol dehydrogenase 5 (RDH5) catalyzes the final oxidation of the visual cycle within the retinal pigment epithelium (RPE), regenerating 11-*cis*-retinal for transport to the photoreceptors. Here, 11-*cis*-retinal combines with opsins to renew the light-accepting ability of the visual pigments. Without 11-*cis*-retinal, the visual pigments (e.g., rhodopsin in rods) are unable to accept light and therefore do not initiate the phototransduction cascade. Loss-of-function mutations in *RDH5* markedly slow regeneration of 11-*cis*-retinal and rhodopsin, manifesting as congenital night blindness. Although initially thought to primarily affect rods, cone dysfunction^1^ and degeneration do occur in patients with *RDH5* mutations,^2^^–^^9^ and this can progress to macular degeneration (MD).^10^^–^^15^ Additionally, patients with *RDH5* mutations typically develop white fundus spots, thus the name fundus albipunctatus.

Although *Rdh5* knockout mice have been generated, they do not recapitulate the human phenotype, having normal rod function and showing delayed rod recovery only after prolonged bleaching.^16^^,^^17^ To develop a better functional model for human *RDH5*-associated retinopathy, *Rdh5*^−/−^*/Rdh11*^−/−^ mice were generated. These mice have a phenotype that more closely resembles *RDH5*-associated retinopathy in humans and have been used to assess potential translational therapies.

Oral supplementation of the double-knockout mice with 9-*cis*-retinal significantly improved rod and cone function.^18^ Based on these findings, supplementation with 9-*cis*-beta carotene has been investigated in clinical trials for *RDH5*-associated retinopathy, although with contradictory results. The trials used oral supplementation with 9-*cis*-beta carotene–rich alga (*Dunaliella bardawil*) powder. Although an initial 3-month-long study showed that therapy resulted in some electroretinogram (ERG) amplitude improvement (Clinicaltrials.gov: NCT00569023),^19^ a longer-term study from Japan (UMIN Clinical Trials Registry R000020490)^20^ showed a detrimental effect on ERG amplitude after 1 year of oral dosing with the *D.* *bardawil* powder. There is therefore an unmet need for an effective therapy for *RDH5*-associated retinopathies.

As previously described, *RDH5*^−/−^ cats, a large-animal model, recapitulate major features of the *RDH5*-associated retinopathy phenotype offering a quality model species for studying potential therapies. Not only do these cats have delayed dark adaptation caused by rod dysfunction, but they also have variability in area centralis (AC) degeneration (analogous to variable human MD).^21^ In addition to rod dysfunction, we previously highlighted the effects of this disease on cones in *RDH5*^−/−^ cats, including delayed cone recovery after exposure to repeated bright flash stimuli. Although all *RDH5*^−/−^ cats exhibit these effects on retinal function, only a subset develops AC degeneration. In the early stages of degeneration, the photoreceptors are lengthened and distorted. With progression this can lead to thinning of the outer nuclear layer (ONL) and degeneration of the photoreceptors of the AC. In some affected cats, this degeneration advances to an almost complete loss of the ONL and photoreceptors.^22^ *RDH5*^−/−^ cats’ AC degeneration and photoreceptor dysfunction are very similar to the features of disease seen in patients with fundus albipunctatus, making them a valuable large-animal model for *RDH5*-associated retinopathies.

*RDH5* ^−/−^ cats present a unique opportunity to develop and test potential gene augmentation therapy treatments. Adeno-associated virus (AAV) vectors offer a safe and efficient technology for delivery of therapeutic genes to the retina, including the ability for precision targeting of specific cell layers. Additionally, the eye offers an immune privileged site potentially reducing adverse immune responses to the viral vector dosing. The first U.S. Food and Drug Administration (FDA)-approved gene therapy product, voroetigene neparvovec-rzyl (Luxturna; Genentech, South San Francisco, CA, USA), is an AAV2 gene therapy for retinal dystrophy due to biallelic mutations in *RPE65*. Clinical trials for gene augmentation therapy for other inherited retinal degenerations are underway (for a review, see McClements et al.^23^). *RDH5*-associated retinopathy is an attractive target for AAV-based gene augmentation therapy for several reasons. The relatively small gene size of *RDH5* cDNA enables it to be packaged into an AAV vector for retinal delivery. The disconnect between impaired rod function without significant rod loss and the slow development of MD provides a wide window of opportunity for therapeutic intervention. We report a successful, potentially translatable gene augmentation therapy in *RDH5*^−/−^ cats that both resolves night blindness and shows potential to halt or slow AC (macular) degeneration.

## Materials and Methods

### Vector Production

Vectors were produced by the University of Florida Vector Core Lab (Powell Gene Therapy Center). Two therapeutic and one reporter gene constructs were used. Initially, a straight AAV2 serotype packaged with feline RDH5 cDNA under control of a vitelliform macular dystrophy (*VMD2*) promoter was tested and failed to result in RDH5 transgene expression in the RPE. A reporter gene construct (*VMD2-*humanized green fluorescent protein [*hGFP*]) package in an AAV2 with three capsid mutations (Y444F+Y500F+Y730F) was tested. For the final therapeutic construct, the AAV2 triple mutant serotype packaged with *VMD2*-f*RDH5* was used.

### Animals

All animal procedures were reviewed and approved by the Michigan State University Institutional Animal Care and Use Committee and adhered to the ARVO Statement for the Use of Animals in Ophthalmic and Vision Research. Cats were housed at the Michigan State University College of Veterinary Medicine vivarium and kept under a 12-hour light/12-hour dark cycle. All cats used in this study originated from a breeding colony described previously.^21^ Two *RDH5*^−/−^ cats and one control cat were injected for a preliminary vector and reporter gene study. Twelve *RDH5*^−/−^ cats were subretinally injected with the triple capsid mutation therapeutic vector at a dose of 5 × 10^11^ vg/mL and followed to assess efficacy of the therapy (Supplementary Tables S1 and S2 provide full injection and procedure information). Of these, two *RDH5*^−/−^ cats were injected with less vector volume to create small blebs targeting the AC with the purpose of assessing the therapeutic effect on degeneration. An additional two *RDH5*^−/−^ cats were injected with the purpose of assessing retinoid differences between treated and untreated regions. Finally, eight *RDH5*^−/−^ cats were injected with larger vector volumes to create large blebs with the purpose of assessing the effect of the therapy on retinal function. Examples of a small and a large bleb can be seen in Supplementary Figure S1. Estimated bleb size was measured in the Heidelberg Engineering optical coherence tomography (OCT) software using the measure function. See tables in the Supplementary Materials for the ERG wild-type (WT) numbers.

### Subretinal Injection

Standard aseptic surgical approaches were utilized for the injection procedure. Pupils were dilated with tropicamide, USP 1% (Falcon Pharmaceuticals, Fort Worth, TX, USA), and corneas were kept hydrated and protected for the duration. The procedure was performed under an operating microscope, and cats were premedicated with ketamine (2–6 mg/kg; MWI Animal Health, Boise, ID, USA) and midazolam (0.2–0.5 mg/kg; Gland Pharma Limited, Telangana, India). They were induced with isoflurane (VetOne, Paris, France) delivered by mask and then intubated and maintained on isoflurane. After positioning under the operating microscope, a region of conjunctiva was reflected to allow placement of a self-sealing 25-gauge sclerotomy port through pars plana. The injection cannula was placed into the vitreous, and the posterior segment was viewed with a corneal-placed vitrectomy lens. The injector was advanced through the scleral port, and under direct visualization the 39-gauge cannula advanced to penetrate the retina and into the subretinal area, where the vector was injected to create a region of retinal detachment (bleb). The port was removed, and the conjunctiva was sutured (6-0 Vicryl; Ethicon, Somerville, NJ, USA). Subconjunctival steroid/antibiotics, dexamethasone (0.2 mg; Bimeda US, Schaumburg, IL, USA), methylprednisolone acetate (Depo-Medrol, 4 mg; Zoetis, Parsippany, NJ, USA), and gentamicin (4 mg; Fresenius Kabi, Bad Homburg, Germany) were injected. Fundus images were collected to document the position of the bleb. One eye was treated, and the other remained an untreated control for comparison in 10 *RDH5*^−/−^ cats. Two *RDH5*^−/−^ cats were treated bilaterally. One eye of two *RDH5*^−/−^ cats was injected with the original AAV2 vector and one control cat for the reporter gene study. Animals were monitored closely until fully recovered from anesthesia, at which time they returned to the animal housing. Oral prednisolone (1 mg/kg twice daily) (MWI Animal Health) was started prior to injection and maintained on a tapering dosage for 10 days. The goal of all subretinal injections was a bleb encompassing the AC; however, as this is a difficult procedure to control direction of bleb expansion, not all cats received a bleb over the AC (Supplementary Table S1).

### Pre- and Post-Injection Follow-Up

Animals underwent baseline in vivo confocal scanning laser ophthalmoscopy (cSLO) and spectral-domain OCT (SD-OCT) imaging (SPECTRALIS HRA+OCT; Heidelberg Engineering, Heidelberg, Germany) pre-injection under general anesthesia, as previously described.^24^ Wide-angle fundus examination images were also obtained at the time of SD-OCT using a Retcam II video fundus camera (Clarity Medical Systems, Pleasanton, CA, USA). We have previously shown that the disease-related changes in *RDH5*^−/−^ cats are bilaterally symmetrical, making the untreated eye of each animal an appropriate control.^21^

The primary outcome measure for this therapeutic was an improvement in the rate of rod recovery following light exposure. This was measured using the rod dark-adaptation ERG protocol. As a secondary outcome measure, animals were investigated using other ERG protocols (bright flash, cone recovery, scotopic, and photopic). Both primary and secondary ERG outcome measures, SD-OCT, and Retcam images were taken at 3 months, 6 months, and 1 year post-injection. The outer nuclear layer (ONL) thickness of the AC was measured at each time point to determine if the therapy had an effect on retinal structure. Three measurements were taken and averaged together for each eye. Only *RDH5*^−/−^ cats with a subretinal bleb over the AC were used to determine whether therapy preserves structure, as the AC is the main area of degeneration.

### Electroretinography

The effect of the gene therapy on retinal functional recovery was assessed by ERG. Animals were dark adapted overnight to get *RDH5*^−/−^ cats to full dark adaptation for a baseline ERG. Pupils were dilated with tropicamide ophthalmic solution (USP 1%). Cats were premedicated with ketamine (2–6 mg/kg) and midazolam (0.2–0.5 mg/kg). Animals were anesthetized and electrodes placed under illumination of a dim red light. Following intubation, animals were maintained with isoflurane. Eyes were positioned in a primary gaze using stay sutures in the perilimbal conjunctiva (4-0 734G PERMA-HAND Silk Suture; Ethicon). Reference platinum needle skin electrodes were placed 5 mm laterally from the lateral canthus, and the ground electrode was placed in-between the shoulder blades. An ERG-Jet monopolar corneal contact electrode (Fabrinal Eye Care, La Chaux-De-Fonds, Switzerland) with a drop of Optixcare Eye Lube Plus (Aventix, Burlington, ON, Canada) was placed on each eye. ERGs were recorded using an Espion E2 or E3 electrophysiology system with a ColorDome Ganzfeld (Diagnosys, Lowell, MA, USA).

#### Recovery of Dark Adaptation

To assess recovery of dark adaptation following therapy, a previously described ERG protocol was utilized.^21^ Following overnight dark adaptation and a baseline dark-adapted rod ERG recording, cats were exposed to a 30-cd/m^2^ rod-suppressing light for 10 minutes. After 10 minutes, the background light stimulus was removed, and the animals were maintained in the dark with periodic rod ERG recordings taken over the course of 100 minutes. Results are reported as a percentage of baseline at the 100-minute post-light exposure time point.

#### Cone ERG With Repeated Strong Light Exposure (Bright Flash and Cone Recovery Protocols)

Cones respond to stronger light stimuli and typically have rapid recovery from a stimulus. We previously showed that a protocol measuring cone function after repeated strong light stimulus showed impairment of cone recovery in the *RDH5*^−/−^ cats.^22^ This protocol consists of alternating flashes of 7.2 cd⋅s/m^2^ from which the ERG is recorded interspersed with strong light exposure consisting of four 1000-cd⋅s/m^2^ flashes. This is repeated with 5 seconds between each group of flashes 25 times in total to assess the impact the therapy had on the accumulative effects of the strong light stimuli. This protocol is referred to as the bright flash protocol, and results are reported as a percentage of bright flash baseline at the final step of the protocol. Immediately following the bright flash protocol, recovery of cone function was assessed by recording ERG responses to 7.2-cd⋅s/m^2^ flashes every 5 minutes over a period of 120 minutes in the presence of a rod-suppressing background light. This protocol is referred to as the cone recovery protocol, and results are reported as a percentage of bright flash baseline at the 120-minute time point.

#### Scotopic and Photopic Comparisons

A standard scotopic dark-adapted luminance series (20 steps ranging from –-4.5 log cd⋅s/m^2^ to 1.36 log cd⋅s/m^2^) was followed by exposure to a 30-cd/m^2^ rod-suppressing white light, a light-adapted luminance series (12 steps ranging from –2.4 to 1.36 log cd⋅s/m^2^) and a 33-Hz 3-cd⋅s/m^2^ flicker as previously described.^25^ Photopic amplitudes and peak times at the 2.482-, 7.2-, and 23-cd⋅s/m^2^ flash strengths and 33-Hz flicker were compared between treated and untreated eyes for analysis.

### Waveform Modeling

The a-wave and b-wave amplitudes and peak times for all ERGs were measured using standard International Society for Clinical Electrophysiology of Vision (ISCEV) protocols.^26^ As previously described, the Naka–Rushton function was used to fit the first limb of the scotopic b-wave luminance-response plot.^22^ Additionally, the leading edge of the scotopic rod a-wave was modeled with flashes ranging from 0.996 to 7.2 cd⋅s/m^2^ using the Birch and Hood version of the Lamb and Pugh rod phototransduction model.^22^

### Statistical Analysis of ERGs

ERG data were tested for normality using a Shapiro–Wilk test. If the data were normally distributed, a paired *t*-test comparing treated and untreated individuals was performed and an unpaired *t*-test was performed between WT versus treated and WT versus untreated. If the data were not normally distributed, a non-parametric Wilcoxon signed rank test was performed. All ERG statistical analyses were performed in RStudio 2023.06.1+524.^27^ Paired and unpaired *t*-tests and Wilcoxon signed-rank tests were performed using the R package stats. The 95% confidence intervals, or the highest attainable confidence interval when 95% confidence intervals could not be calculated, were reported when possible. Due to low sample size, zero differences, and tied ranks in paired comparisons, confidence intervals were not always estimable. Animals with small blebs were excluded from ERG functional statistical comparisons due to limited retinal treatment regions. Additionally, animals injected in both eyes (no control eye) were excluded from ERG comparisons. Effect size for parametric data was calculated using Hedges’ *g*, and nonparametric data used matched-pairs rank biserial correlation. Effect size was calculated using the effectsize package in RStudio.^28^

### Immunohistochemistry and Western Blot

Following humane euthanasia, eyes were removed and fixed in 4% paraformaldehyde (PFA; Electron Microscopy Sciences, Hatfield, PA, USA) in phosphate-buffered saline (PBS; Sigma-Aldrich, St. Louis, MO, USA) and embedded in optimal cutting media (TissueTek OCT; Electron Microscopy Sciences) for frozen sectioning as previously described.^29^ For IHC, 14-µm cryosections through the treated retina were used. A RDH5 rabbit primary antibody (NBP2-15097; Novus Biologicals, Centennial, CO, USA) at a concentration of 1:100 was used, and an Invitrogen Alexa Fluor 488 goat anti-rabbit secondary antibody was used at a concentration of 1:500 (A11008; Thermo Fisher Scientific, Waltham, MA, USA). All slides were imaged with an Eclipse 80i fluorescence microscope (Nikon Corporation, Tokyo, Japan) with a pco.panda 4.2 sCMOS camera (70108074001; Excelitas, Pittsburgh, PA, USA).

For western blotting, retina RPE choroid was dissected and placed in 400-µL radioimmunoprecipitation assay (RIPA) buffer with proteinase inhibitors and processed as previously described.^21^ However, the RDH5 antibody was diluted to 1:500, α-tubulin was diluted to 1:2000, and a chemiluminescent substrate was used (34076; Thermo Fisher Scientific). Retina RPE choroid was obtained from the treated bleb region and the untreated region of the same eye (*n* = 1). Retina RPE choroid from one WT cat was collected. Western blotting was quantified by densitometric analysis using ImageJ (National Institutes of Health, Bethesda, MD, USA).^30^ RDH5 protein was normalized to the corresponding loading control (α-tubulin), and the RDH5 protein/α-tubulin ratio was calculated.

### Resin-Embedded Semi-Thin Sections

Following humane euthanasia, eyes were removed and fixed in 3% glutaraldehyde and 2% paraformaldehyde in 0.1-M sodium cacodylate buffer (Electron Microscopy Sciences), as previously described.^25^ Following fixation, 3-mm-diameter biopsy punches were collected from the AC of treated and untreated eyes of two *RDH5*^−/−^ cats with preservation of structure. Color fundus images of the eyes showing the injection bleb and vascular pattern to allow identification of the AC were used to guide tissue collection. The biopsy punches were resin embedded as previously described.^25^ Then, 1000-nm-thick sections were obtained and stained with epoxy tissue stain (Electron Microscopy Sciences) and imaged on a Nikon Eclipse 80i light microscope.

### Retinoid Extraction and Analysis

*RDH5* ^−/−^ (*n* = 2) and *RDH5^+/+^* (*n* = 1) cats were dark adapted overnight, followed by brief exposure to light (30 cd/m^2^ for 10 minutes) and 100 minutes of dark adaptation prior to euthanasia. Eyes were enucleated under dim light, and biopsies were acquired from treated and untreated sections with the retina intact (containing RPE and choroid). Retinal punches were flash frozen and stored at –80°C. Retinoid analysis procedures were all performed under dim red light.

For retinoid extraction, samples were transferred to an ice-cold buffered solution consisting of 10-mM sodium phosphate (pH 8.0), 100-mM of hydroxylamine (Sigma-Aldrich), and 50% v/v methanol (Sigma-Aldrich); homogenized with a tissue grinder; and incubated at 22°C for 15 minutes. Next, 3 mL of 100% ethyl acetate (Thermo Fisher Scientific) and 2 mL of 3-M NaCl were added to the samples. The mixture was vigorously shaken and then centrifuged to promote phase separation, and the upper organic layer was collected into a borosilicate tube. An additional 3 mL of ethyl acetate was added and the extraction procedure repeated. The combined organic layers, containing extracted retinoids, were dried in vacuo. The dried sample was reconstituted in 450 µL of 100% hexanes (Thermo Fisher Scientific), and a 100 µL portion was analyzed by normal phase high-performance liquid chromatography (HPLC; 1260 Infinity II LC system equipped with a diode array detector; Agilent Technologies, Santa Clara, CA, USA) using a Zorbax Rx-SIL column (5 µm, 4.6  ×  250 mm; Agilent Technologies). A stepwise elution protocol was used at a solvent flow rate of 1.4 mL/min, first with 0.6% v/v ethyl acetate in hexanes for 17 minutes followed by 10% v/v ethyl acetate in hexanes for 45 minutes. Retinoid elution was monitored at 325 nm and 360 nm. Retinoid peak areas were integrated using ChemStation software (Agilent Technologies). Retinoid standard curves allowed conversion of 11-*cis*-retinal oxime and retinyl ester peak areas to absolute masses. Raw chromatograms and quantified retinoid species were plotted and analyzed with Prism (GraphPad Software, Boston, MA, USA).

## Results

### AAV2-VMD2-fRDH5 Does Not Result in Transgene Expression

The initial construct with a native AAV2 capsid was injected subretinally in one eye of two *RDH5*^−/−^ cats. No ERG rescue was detected nor expression of RDH5 in the retina (data not shown).

### Vector With an AAV2 Triple Capsid Mutation Drives Expression in RPE With Improvement in the Classic Visual Cycle

To confirm the AAV2 triple mutant capsid along with the *VMD2* promoter could drive RPE expression of the transgene, an initial reporter gene (*hGFP*) study was performed using a control cat. This confirmed that the *GFP* transgene was present and specific to the RPE (Supplementary Fig. S2). Immunohistochemistry and western blotting of the treated eyes confirmed that expression of the *RDH5* transgene occurred exclusively in the RPE of treated regions of the *RDH5*^−/−^ cats (Fig. 1, Supplementary Fig. S3). HPLC demonstrated that the expressed RDH5 was functional. Retinal samples were collected after a brief photobleach followed by dark adaptation for 100 minutes. The control samples from WT cats showed the presence of 11-*cis*-retinal, but the treated retinal regions from *RDH5*^−/−^ cats showed decreased all-*trans*-retinyl esters and increased 11-*cis*-retinal formation compared to untreated regions (Fig. 2, Supplementary Fig. S4, Supplementary Table S3). The abnormal presence of 13-*cis*-retinyl esters associated with RDH5 loss of function^21^ was also corrected by the gene therapy. Together, the data demonstrate that the gene therapy corrected the biochemical defect in the visual cycle associated with RDH5 loss of function.

**Figure 1.**
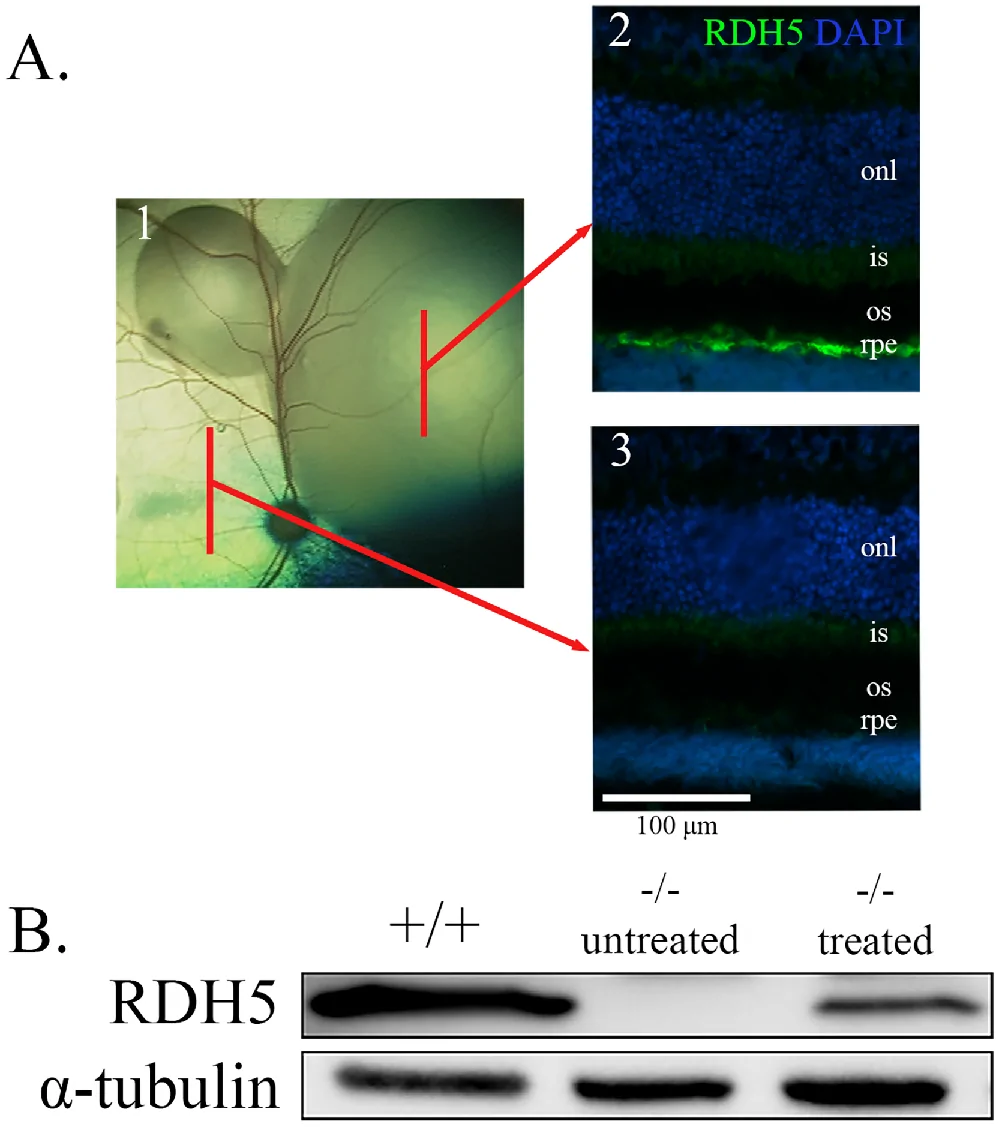
Subretinal injection of AAV2(triple Y>F)-*VMD2-fRDH5* vector in a *RDH5*^−/−^ cat*.* (**A1**) Post-injection fundus image showing the subretinal blebs (*RDH5*^−/−^ cat #18-142). (**A2**) IHC from injected region showing RDH5 expression in RPE at 6 months post-injection. (**A3**) IHC from the non-injected region, with no RDH5 expression detectable in RPE. (**B**) Western blot comparing RDH5 and α-tubulin among *RDH5^+/+^*, *RDH5*^−/−^ untreated, and *RDH5*^−/−^ treated regions. Normalized RDH5 protein expression: +/+ 1.00, –/– untreated 0.02, and –/– treated 0.34.Treated and untreated samples were obtained from the same eye. onl, outer nuclear layer; is, photoreceptor inner segment; os, photoreceptor outer segment; rpe, retinal pigment epithelium.

**Figure 2.**
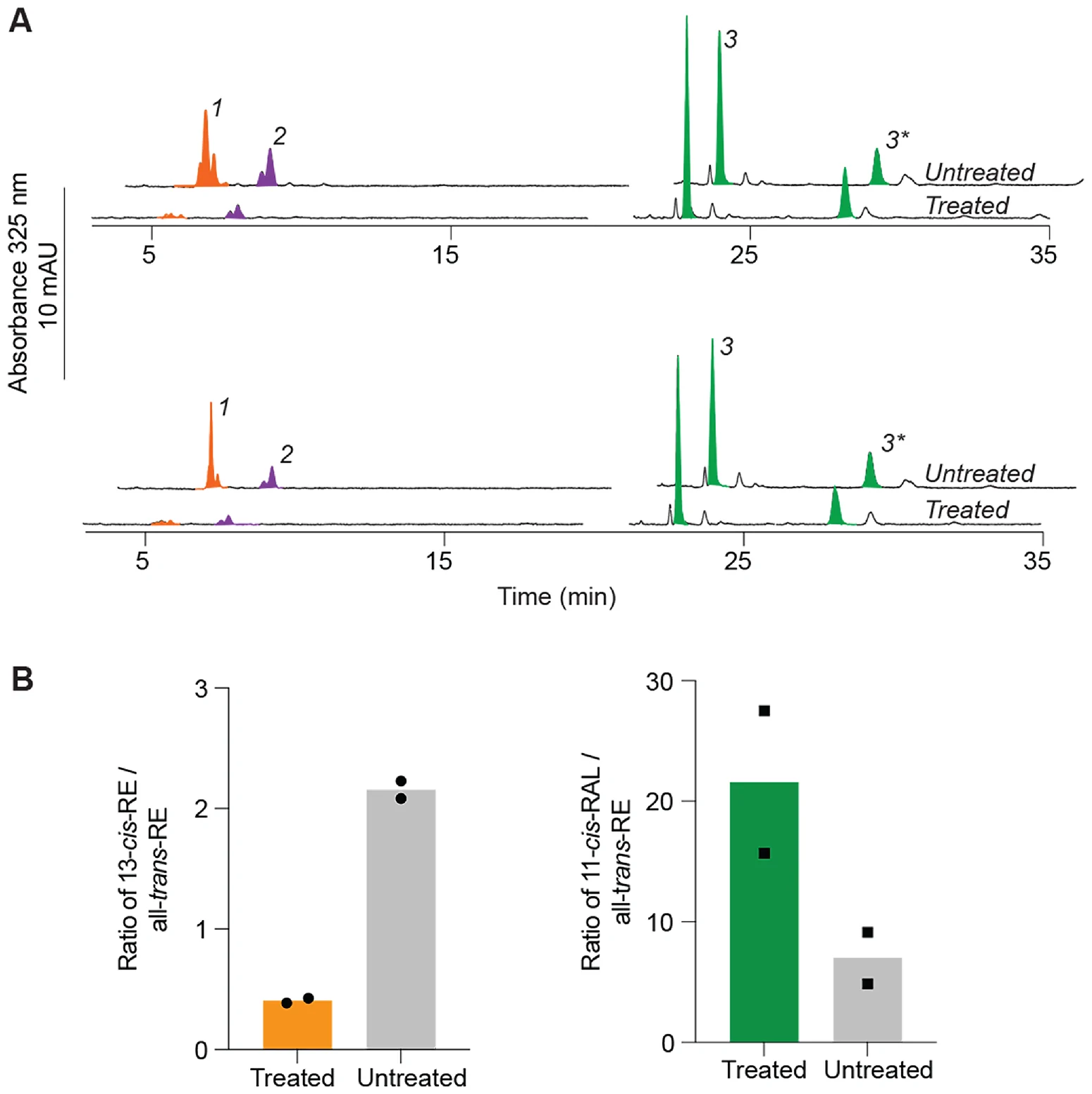
Retinoid analyses from *RDH5*^−/−^ cat retinas following gene therapy*.* (**A**) HPLC chromatograms (at 325-nm wavelength) of retinoids extracted from retinas of two *RDH5*^−/−^ cats injected with *RDH5*-AAV. Retinal punches were acquired following a brief period of photobleach followed by dark adaptation. Retinoids were analyzed from treated and untreated retina sections of each eye. In both cat retinas, the 11-*cis*-retinaldehyde (*syn-* and *anti*-oximes; *green*; denoted as *3* and 3*) peak area was observed to be larger in treated samples compared to their untreated counterparts. Similarly, peak areas for 13-*cis*-retinyl and all-*trans*-retinyl esters (*1*, *orange*; *2*, *purple*, respectively) were observed to be greater in the untreated samples compared to treated. (**B**) Comparison of the quantitative mass ratios of 13-*cis*-retinyl esters and all-*trans*-retinyl esters (*left*) as well as 11-*cis*-retinaldehyde and all-*trans*-retinyl esters (*right*) between treated (*orange* and *green bars*) and untreated (*gray bars*) samples from the two *RDH5*^−/−^ cats. A gap was introduced in the chromatogram traces to remove a solvent change artifact occurring between the two isocratic elution steps.

### Gene Therapy Improves Photoreceptor Recovery Following Light Exposure

Scotopic ERGs following overnight dark adaptation were comparable between *RDH5*^−/−^ and WT cats with only a few minor changes in certain parameters, as we have previously reported.^22^ In the current study, we found very few differences in Naka–Rushton and a-wave modeling parameters following overnight dark adaptation between treated and untreated eyes (the differences are highlighted in Supplementary Table S4). Similarly, for regular photopic ERG, we previously reported little difference between *RDH5*^−/−^ and WT cats,^22^ and, not surprisingly, the difference between treated and untreated eyes herein was negligible (Supplementary Table S5).

Following a bleach, the *RDH5*^−/−^ cats showed very little recovery of rod function as assessed by ERG over a 100-minute period compared to almost full recovery in WT cats. The degree of rod recovery (Fig. 3) was significantly improved in gene therapy–treated eyes compared to untreated eyes (3 months, *P* < 0.001; 6 months, *P* = 0.008; 1 year, *P* = 0.03) (see Supplementary Table S6 for the full statistical report). Effect size analysis indicated large treatment effects, with Hedges’ *g* values of 2.21 at 3 months and 1.18 at 1 year post-injection, and a rank-biserial correlation of 1.00 at 6 months post-injection. As only part of the retina was treated, the fullfield flash ERG, which records from the entire retina (treated and untreated), was not expected to show rod functional recovery comparable to that in WT cats.

**Figure 3.**
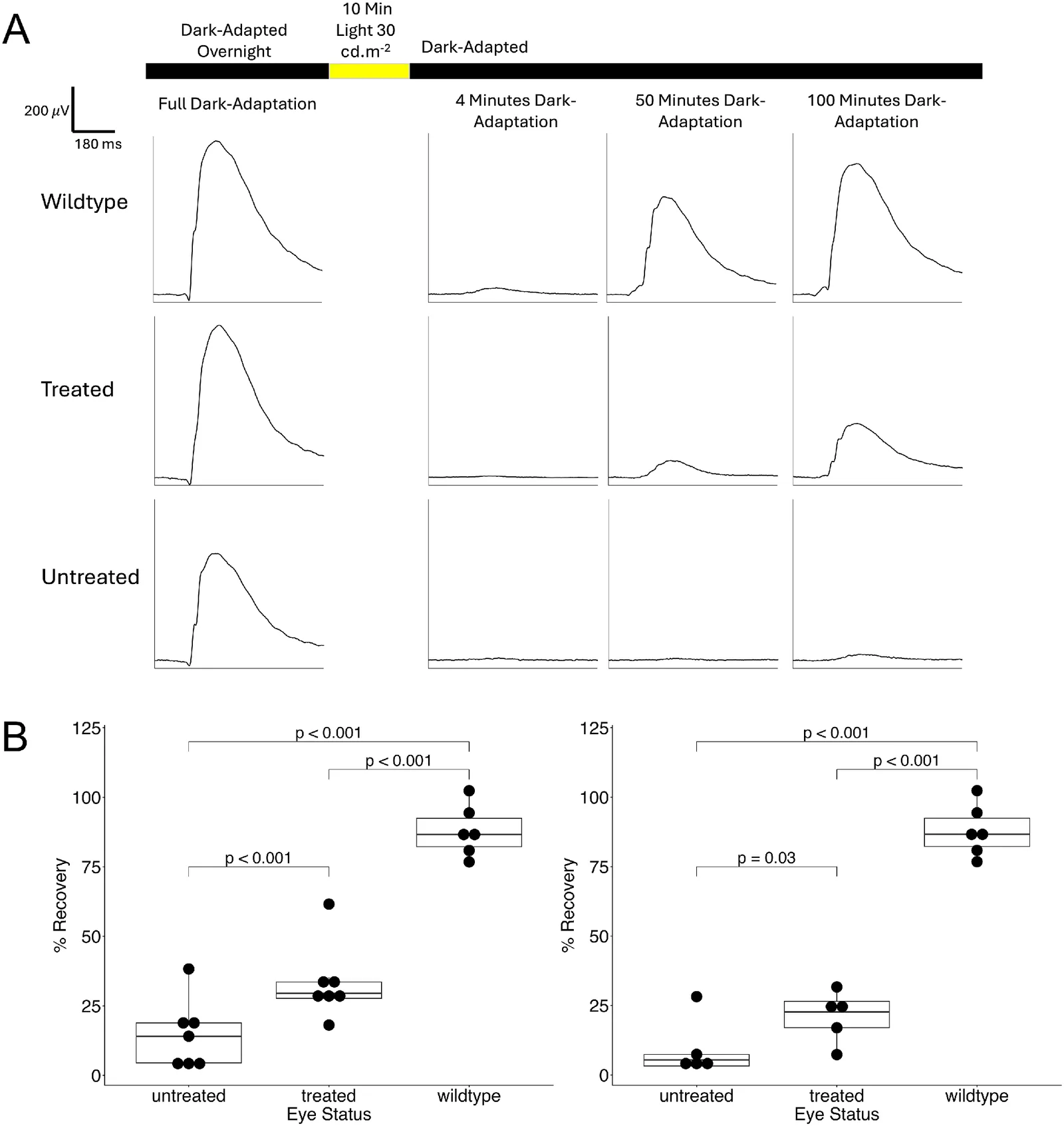
Recovery of dark adaptation in *RDH5*^−/−^ treated versus untreated eyes versus wild-type*.* (**A**) Representative ERG waveforms from each group at baseline (overnight dark adaptation) and at 4, 50, and 100 minutes after light exposure. The representative waveform is from treated *RDH5*^−/−^ cat #18-142. (**B**) Percent rod recovery comparisons 3 months after gene therapy (*RDH5*^−/−^, *n* = 7; WT, *n* = 6) (*left*) and rod percent recovery comparisons 1 year after gene therapy (*RDH5*^−/−^, *n* = 5; WT, *n* = 6) (*right*). Treated versus untreated data were analyzed using paired *t*-tests, and the treated versus wild-type and untreated versus wild-type were analyzed using unpaired *t*-tests.

In addition to a delay in rod photoreceptor recovery from light exposure, we assessed the ability of the cones in treated *RDH5*^−/−^ cats to recover following a protocol involving repeated exposure to strong flashes of light with a regular ERG recording between strong flashes. The previously described protocol was termed the bright flash protocol. Over the period of repeated flash exposures, there was a decline in the photopic ERG amplitudes. There was no significant difference between these ERG amplitudes between treated and untreated eyes (Supplementary Table S7). Following this suppression of cone function, the recovery of cone responses was followed over the subsequent 120 minutes with no further strong light stimulus. This protocol was termed the cone recovery protocol. The treated eyes had significantly greater recovery of b-wave amplitude assessed as a percentage of bright flash baseline at all post-injection time points (3 months, 0.007; 6 months, 0.006; 1 year, 0.03) compared to their untreated counterparts (Fig. 4). Effect size analysis indicated large treatment effects, with Hedges’ *g* values of 1.50 at 3 months, 1.38 at 6 months, and 1.82 at 1 year post-injection. There was no significant difference in the a-wave percentage of baseline between treated and untreated eyes at any time points (Supplementary Table S7).

**Figure 4.**
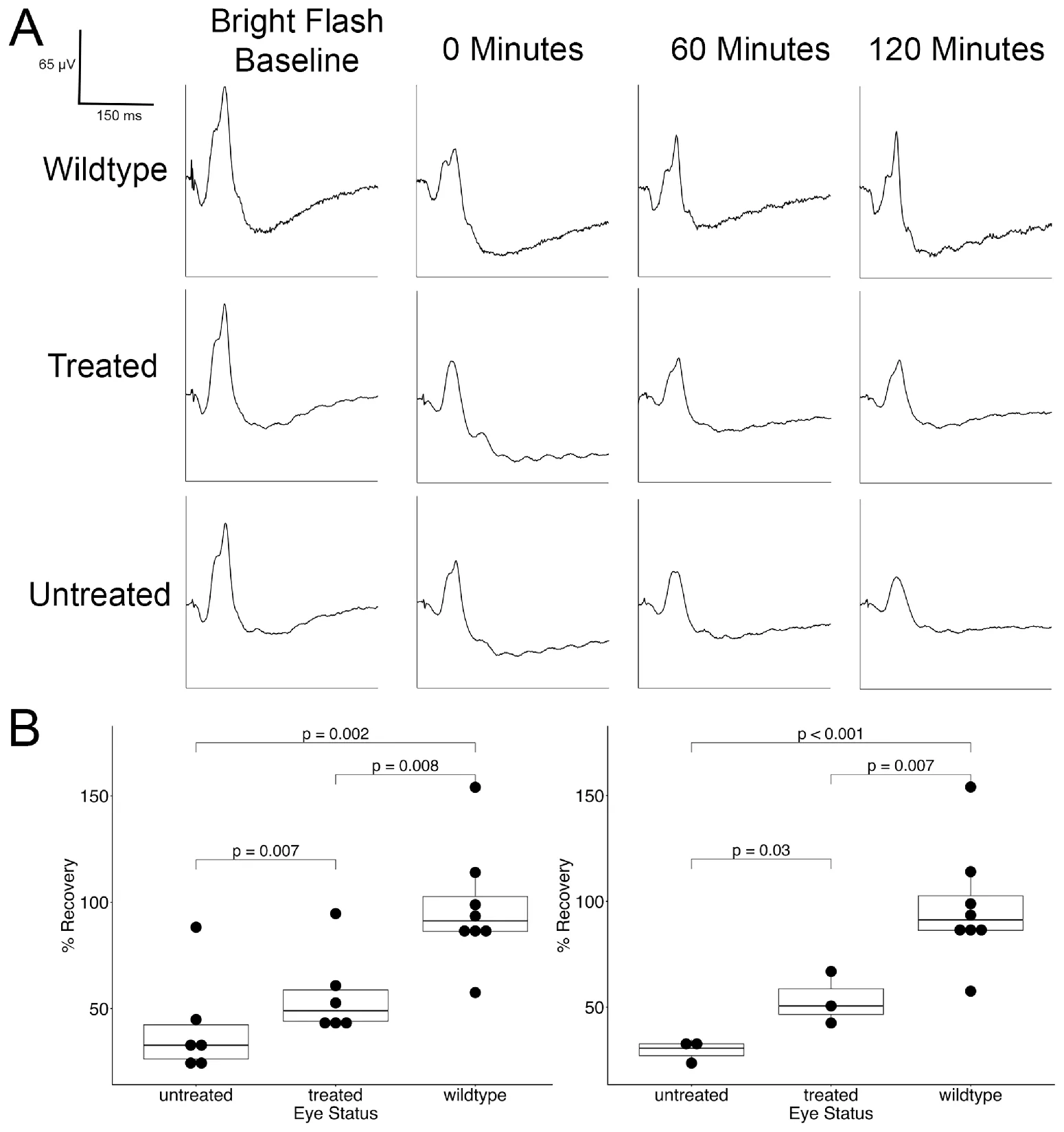
Cone recovery ERG for treated versus untreated *RDH5*^−/−^ eyes versus wild-type*.* (**A**) Average waveforms from 6 months post-injection for treated and untreated groups at the bright flash baseline and at 0, 60, and 120 minutes. Wild-type is a representative waveform. (**B**) Three-month b-wave percent recovery comparisons at 120 minutes (*RDH5*^−/−^, *n* = 6; WT, *n* = 8) (*left*) and 1 year after gene therapy comparing b-wave percent recovery at 120 minutes (*RDH5*^−/−^, *n* = 3; WT, *n* = 8) (*right*). Treated versus untreated data were analyzed using paired *t*-tests, and the treated versus wild-type and untreated versus wild-type data were analyzed using unpaired *t*-tests. CR, cone recovery.

### Therapy Provides Structural Preservation of the AC

In addition to functional improvement resulting from the gene augmentation therapy, *RDH5*^−/−^ cats were assessed for structural preservation using SD-OCT imaging. A subset of *RDH5*^−/−^ cats developed a bilaterally symmetrical progressive outer retinal thinning in the AC. Two cats in which the injection bleb encompassed the AC developed AC degeneration in the untreated control eye. Although both animals showed clear evidence of structural preservation in the AC region of the treated eye, the degree of preservation differed. In one *RDH5*^−/−^-treated cat (RSA 20-053) there was virtually no degeneration in the AC of the treated region compared to the AC of the untreated region. In the other *RDH5*^−/−^-treated cat (RSA 20-054), there was slowing, but not halting, of the degeneration in the treated eye. This structural preservation could also be seen in semi-thin electron microscopy sections across the AC in both *RDH5*^−/−^ cats (Fig. 5).

**Figure 5.**
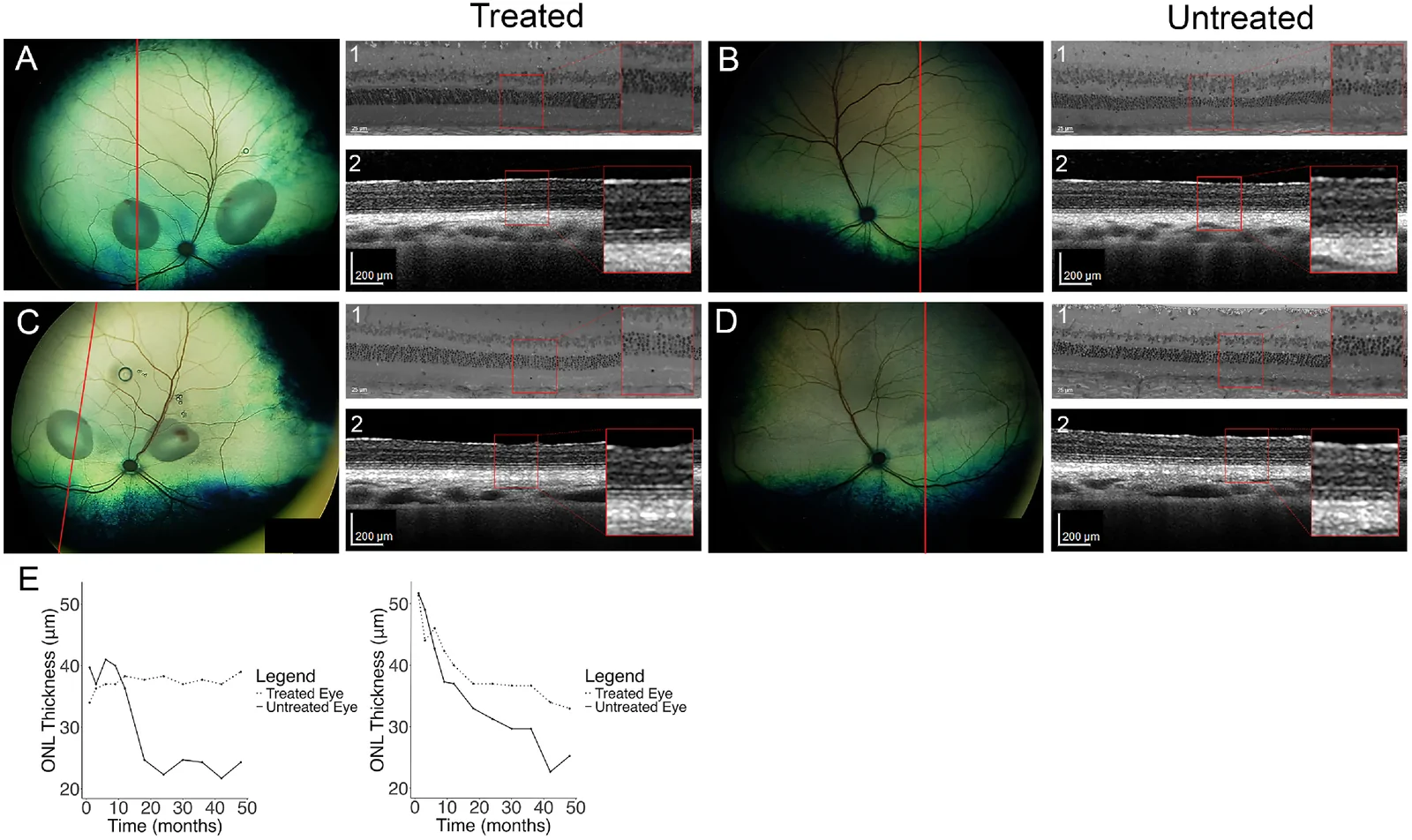
Structural preservation resulting from gene therapy*.* (**A**) RetCam image of *RDH5*^−/−^ cat #20-053 showing treated bleb regions. Treated retina at the AC can be seen on both semi-thin sections (**1**) and SD-OCT (**2**). (**B**) Retcam image of the untreated eye of *RDH5*^−/−^ cat #20-053. Untreated retina at the AC can be seen on both semi-thin sections (**1**) and SD-OCT (**2**). (**C**) Retcam image of *RDH5*^−/−^ cat #20-054 showing treated bleb regions. Treated retina at the AC can be seen on both semi-thin sections (**1**) and SD-OCT (**2**). (**D**) Retcam image of the untreated eye of *RDH5*^−/−^ cat #20-054. Untreated retina at the AC can be seen on both semi-thin sections (**1**) and SD-OCT (**2**). Red lines on RetCam images indicate where semi-thin and OCT images originate from. All semi-thin sections are imaged at 10× magnification. (**E**) Comparison of ONL thickness over time at the AC between treated (*dotted line*) and untreated (*solid line*) in *RDH5*^−/−^ cat #20-053 (*left*) and cat #20-054 (*right*). OCT images and samples for semi-thins from both *RDH5*^−/−^ cats were collected at 4 years post-injection.

### Adverse Effects

The injections were well tolerated with no inflammation beyond the typical changes seen as a result of a subretinal injection. However, incipient and non-progressive cataracts developed in two of the treated eyes. One developed the cataract 3 months post-injection, and the other developed the cataract 1 year post-injection. It is unclear if the cataract developed as a direct result of treatment. Beyond the scar from the injection retinotomy, there was no retinal degeneration associated with the treated bleb region (Supplementary Fig. S5).

## Discussion

There is currently an unmet need for therapy for patients with *RDH5*-associated retinopathies. Although the night-blindness phenotype may be manageable, the potential of progressive MD represents a threat to the high-acuity vision needed for normal activities of daily living. AAV gene augmentation is proposed to be an appropriate approach. AAV vectors are being used in many clinical trials with promising results,^23^ and an FDA therapy for treating lack of function of another RPE visual cycle gene, *RPE65*, has been approved.^31^ The therapy should be safe and improve the supply of 11-*cis*-retinal to photoreceptors, ideally providing improved rod photoreceptor recovery and protecting the cones and macula from degeneration. Here, we have reported a promising AAV therapy that provides a proof of concept for treating *RDH5* retinopathies.

We identified an AAV serotype/promoter combination that efficiently transduced feline RPE from a subretinal injection using a reporter gene study. This was an AAV2 vector with a triple capsid Y>F modification coupled with a *VMD2* promoter. A therapeutic construct using this serotype/promoter combination was constructed to deliver feline *RDH5* cDNA. IHC and western blotting confirmed that this therapeutic construct resulted in *RDH5* expression just in the region of the subretinal bleb, as expected. HPLC analysis of treated retinal samples showed clearance of abnormal 13-*cis*-retinyl esters and improved conversion of 11-*cis*-retinol to the 11-*cis*-retinal required for rhodopsin regeneration. This was reflected in an improvement in rod ERG recovery following bleaching.

Significant improvement in the percent of b-wave recovery after cone functional suppression from repeated bright-light exposure (cone recovery protocol) between treated and untreated eyes indicated that RDH5 supplementation also improves cone functionality. The untreated eyes had a very reduced 11-*cis*-retinal supply from the classic RPE visual cycle, likely meaning they rely on the intraretinal visual cycle to allow reactivation of the cone opsins. With continued strong light exposure, it is likely that the 11-*cis*-retinal supply from the intraretinal visual cycle will become depleted. With RDH5 supplementation, there is more 11-*cis*-retinal available via the classic visual cycle for the cones to recover more quickly than those in the untreated *RDH5*^−/−^ retina. Therapy is expected to improve 11-*cis*-retinal supply from the RPE visual cycle but will have no effect on the intraretinal visual cycle.

Although treatment resulted in significant differences between the treated and untreated eyes for some ERG parameters, treated eyes did not reach WT control levels for these parameters. Treatment blebs encompassed only a small portion of the retina, and all ERGs used were fullfield flash with a Ganzfeld with summated input from the entire retina, treated and untreated. Therefore, even with optimal transduction within the treatment bleb responses are lower than for normal WT retina.

Structural ONL preservation in the AC was seen in the two *RDH5*^−/−^-treated cats in which the AC was successfully targeted and which developed AC degeneration in the untreated eye. It can sometimes be difficult to predict the direction in which the injection bleb extends, meaning that even when the intention was to include the AC the bleb did not necessarily extend to include it. Furthermore, not all the *RDH5*^−/−^ cats in the study developed AC degeneration. Of the two cats that had therapy that included the AC and showed AC degeneration in the untreated eye, the AC degeneration was slowed in one cat and halted in the other. These cats were followed for 4 years. The difference in degree of preservation of the AC between these two *RDH5*^−/−^ cats could reflect differences in RPE transduction efficiency between the two cats or factors such as background genetics. These results are preliminary, and further studies are required to investigate the photoreceptor preservation from therapy and understand the differences in structural rescue.

This gene augmentation therapy treatment improves functional rod and cone recovery and also shows promise in being able to slow and potentially halt AC degeneration in *RDH5*^−/−^ cats. Future directions of this therapy include continued long-term investigation into whether the AC degeneration is halted or just slowed by treatment. In conclusion, this preliminary study showed that *RDH5* AAV2 gene augmentation therapy offers a promising and potentially translatable therapy able not only to improve retinal function but also to slow the debilitating MD phenotype seen in a subset of humans with *RDH5*-associated retinopathies.

## Supplementary Material

Supplement 1

## References

[1] Cideciyan AV, Haeseleer F, Fariss RN, et al. Rod and cone visual cycle consequences of a null mutation in the 11-cis-retinol dehydrogenase gene in man. *Vis Neurosci*. 2000; 17(5): 667–678. 10.1017/s0952523800175029 PMC1410813 11153648

[2] Makiyama Y, Ooto S, Hangai M, et al. Cone abnormalities in fundus albipunctatus associated with RDH5 mutations assessed using adaptive optics scanning laser ophthalmoscopy. *Am J Ophthalmol*. 2014; 157(3): 558–570. 10.1016/j.ajo.2013.10.021 24246574

[3] Querques G, Carrillo P, Querques L, Bux AV, Del Curatolo MV, Delle Noci N. High-definition optical coherence tomographic visualization of photoreceptor layer and retinal flecks in fundus albipunctatus associated with cone dystrophy. *Arch Ophthalmol*. 2009; 127(5): 703–706. 10.1001/archophthalmol.2009.87 19433727

[4] Sergouniotis PI, Sohn EH, Li Z, et al. Phenotypic variability in RDH5 retinopathy (fundus albipunctatus). *Ophthalmology*. 2011; 118(8): 1661–1670. 10.1016/j.ophtha.2010.12.031 21529959

[5] Kuehlewein L, Nasser F, Gloeckle N, Kohl S, Zrenner E. Fundus albipunctatus associated with cone dysfunction. *Retin Cases Br Rep*. 2017; 11: S73–S76. 10.1097/ICB.0000000000000420 27627638

[6] Miyake Y, Shiroyama N, Sugita S, Horiguchi M, Yagasaki K. Fundus albipunctatus associated with cone dystrophy. *Br J Ophthalmol*. 1992; 76(6): 375–379. 10.1136/bjo.76.6.375 PMC504291 1622952

[7] Nakamura M, Hotta Y, Tanikawa A, Terasaki H, Miyake Y. A high association with cone dystrophy in fundus albipunctatus caused by mutations of the RDH5 gene. *Invest Ophthalmol Vis Sci*. 2000; 41(12): 3925–3932. 11053295

[8] Nakamura M, Lin J, Miyake Y. Young monozygotic twin sisters with fundus albipunctatus and cone dystrophy. *Arch Ophthalmol*. 2004; 122(8): 1203–1207. 10.1001/archopht.122.8.1203 15302662

[9] Rüther K, Janssen B, Kellner U, et al. Clinical and genetic findings in a patient with fundus albipunctatus. *Ophthalmologe*. 2004; 101(2): 177–185. 10.1007/s00347-003-0895-y 14991316

[10] Schatz P, Preising M, Lorenz B, et al. Lack of autofluorescence in fundus albipunctatus associated with mutations in RDH5. *Retina*. 2010; 30(10): 1704–1713. 10.1097/IAE.0b013e3181dc050a 20829743

[11] Yamamoto H, Yakushijin K, Kusuhara S, Escaño MFT, Nagai A, Negi A. A novel RDH5 gene mutation in a patient with fundus albipunctatus presenting with macular atrophy and fading white dots. *Am J Ophthalmol*. 2003; 136(3): 572–574. 10.1016/s0002-9394(03)00332-5 12967826

[12] Nakamura M, Skalet J, Miyake Y. RDH5 gene mutations and electroretinogram in fundus albipunctatus with or without macular dystrophy. *Doc Ophthalmol*. 2003; 107(1): 3–11. 10.1023/a:1024498826904 12906118

[13] Ajmal M, Khan MI, Neveling K, et al. Novel mutations in RDH5 cause fundus albipunctatus in two consanguineous Pakistani families. *Mol Vis*. 2012; 18: 1558–1571. PMC3380946 22736946

[14] Hotta K, Nakamura M, Kondo M, et al. Macular dystrophy in a Japanese family with fundus albipunctatus. *Am J Ophthalmol*. 2003; 135(6): 917–919. 10.1016/s0002-9394(02)02290-0 12788147

[15] Pras E, Pras E, Reznik-Wolf H, et al. Fundus albipunctatus: novel mutations and phenotypic description of Israeli patients. *Mol Vis*. 2012; 18: 1712–1718. PMC3399783 22815624

[16] Driessen CA, Janssen BP, Winkens HJ, et al. Null mutation in the human 11-cis retinol dehydrogenase gene associated with fundus albipunctatus. *Ophthalmology*. 2001; 108(8): 1479–1484. 10.1016/s0161-6420(01)00640-6 11470705

[17] Shang E, Lai K, Packer AI, et al. Targeted disruption of the mouse cis-retinol dehydrogenase gene: visual and nonvisual functions. *J Lipid Res*. 2002; 43(4): 590–597. 11907141

[18] Maeda A, Maeda T, Palczewski K. Improvement in rod and cone function in mouse model of fundus albipunctatus after pharmacologic treatment with 9-cis-retinal. *Invest Ophthalmol Vis Sci*. 2006; 47(10): 4540–4546. 10.1167/iovs.06-0215 PMC1764978 17003450

[19] Rotenstreich Y, Harats D, Shaish A, Pras E, Belkin M. Treatment of a retinal dystrophy, fundus albipunctatus, with oral 9-cis-β-carotene. *Br J Ophthalmol*. 2010; 94(5): 616–621. 10.1136/bjo.2009.167049 19955196

[20] Mizobuchi K, Hayashi T, Ueno S, et al. One-year outcomes of oral treatment with alga capsules containing low levels of 9-cis-β-carotene in RDH5-related fundus albipunctatus. *Am J Ophthalmol*. 2023; 254: 193–202. 10.1016/j.ajo.2023.06.013 37343743

[21] Occelli LM, Daruwalla A, De Silva SR, et al. A large animal model of RDH5-associated retinopathy recapitulates important features of the human phenotype. *Hum Mol Genet*. 2021; 31(8): 1263–1277. 10.1093/hmg/ddab316 PMC9029234 34726233

[22] Ford LM, Occelli LM, Enfield ME, et al. Functional and structural characterization of a large animal model of RDH5-associated retinopathy. *Transl Vis Sci Technol*. 2026; 15(8): 2. 10.1167/tvst.15.8.2 PMC13450645 42554417

[23] McClements ME, Elsayed MEA, Major L, de la Camara CM-F, MacLaren RE. Gene therapies in clinical development to treat retinal disorders. *Mol Diagn Ther*. 2024; 28(5): 575–591. 10.1007/s40291-024-00722-0 PMC11349810 38955952

[24] Petersen-Jones SM, Occelli LM, Winkler PA, et al. Patients and animal models of CNGβ1-deficient retinitis pigmentosa support gene augmentation approach. *J Clin Invest*. 2018; 128(1): 190–206. 10.1172/JCI95161 PMC5749539 29202463

[25] Occelli LM, Tran NM, Narfström K, Chen S, Petersen-Jones SM. Crx^Rdy^ cat: a large animal model for CRX-associated Leber congenital amaurosis. *Invest Ophthalmol Vis Sci*. 2016; 57(8): 3780–3792. 10.1167/iovs.16-19444 PMC4960999 27427859

[26] Robson AG, Frishman LJ, Grigg J, et al. ISCEV Standard for full-field clinical electroretinography (2022 update). *Doc Ophthalmol*. 2022; 144(3): 165–177. 10.1007/s10633-022-09872-0 PMC9192408 35511377

[27] The R Foundation. The R project for statistical computing. Available at: http://www.R-project.org/. Accessed October 2, 2026.

[28] Ben-Shachar MS, Lüdecke D, Makowski D. Effectsize: estimation of effect size indices and standardized parameters. *J Open Source Software*. 2020; 5(56): 2815.

[29] Mowat FM, Breuwer AR, Bartoe JT, et al. RPE65 gene therapy slows cone loss in Rpe65-deficient dogs. *Gene Ther*. 2013; 20(5): 545–555. 10.1038/gt.2012.63 22951453

[30] Schneider CA, Rasband WS, Eliceiri KW. NIH Image to ImageJ: 25 years of image analysis. *Nat Methods*. 2012; 9(7): 671–675. 10.1038/nmeth.2089 PMC5554542 22930834

[31] Russell S, Bennett J, Wellman JA, et al. Efficacy and safety of voretigene neparvovec (AAV2-hRPE65v2) in patients with RPE65-mediated inherited retinal dystrophy: a randomised, controlled, open-label, phase 3 trial. *Lancet*. 2017; 390(10097): 849–860. 10.1016/S0140-6736(17)31868-8 PMC5726391 28712537

