# Supplementary material for: AAV Gene Augmentation Therapy Rescues the Phenotype in the RDH5−/− Cat Model of Fundus Albipunctatus
Source: Invest Ophthalmol Vis Sci. 2026 Oct 21;67(12):45. doi: 10.1167/iovs.67.12.45 (PMC13712387; doi:10.1167/iovs.67.12.45)
Supplement: Supplementary file 1 [file iovs-67-12-45_s001.docx]

***Figure S1. Bleb size examples.*** An example of (A) two small blebs (*RDH5^-/-^* cat #20-053) and (B) one large and one smaller bleb *(RDH5^-/-^* cat #19-117) immediately post-injection.

***Figure S2. Subretinal injection of AAV2(triple Y>F)-VMD2-hGFP vector in a wild-type cat*. (A)** Post injection fundus image showing subretinal bleb. The red line indicates region of the section (control cat #15-024). **(B)** IHC across injected bleb region, GFP expression in the RPE in the bleb region and no expression outside of bleb. Red arrowhead indicates junction between treated and untreated region. onl, outer nuclear layer; is, inner segment; os, outer segment; rpe, retinal pigment epithelium.

***Figure S3. RDH5 expression is exclusive to the RPE.*** Immunohistochemistry highlighting the presence of RDH5 (polyclonal rabbit, Novus Bio: NBP2-15097, 1:100) in the wildtype and *RDH5^-/-^* treated cat, but not in the *RDH5^-/-^* untreated cat. RPE65 (monoclonal mouse, Dr. Debra Thompson, Kellogg Eye Center, 1:500), an RPE marker, is present in all samples. *RDH5^-/-^* cat # 24-015 at 1-year post-injection.

***Figure S4. Raw chromatograms of retinoids extracted from RDH5^-/-^ cats after treatment with AAV***. (Top panels) Elution profiles of retinoids from treated and untreated retina sections of three cats (A & B are *RDH5^-/-^* and C is *RDH5^+/+^*) by normal-phase HPLC (top panel). A standard for retinyl palmitate was included for identification of retinyl esters. For each animal, elution traces between treated and untreated sections were aligned and retinoid peaks were compared using a dashed line (---). Absorbance peaks are shown at wavelength 325 nm. Note that the B-OD UT had low levels of all retinoids likely due to a smaller than expected size of tissue used for analysis or incomplete retinoid extraction. The B-OD samples were therefore not used for detailed analyses. (Bottom panels) Absorption spectra and their maxima were obtained from the chromatogram peaks associated with each retinoid (labeled *1*, *2*, *3* and *3**; depicting 13-*cis*-retinyl esters, all-*trans*-retinyl esters, *syn*-11-*cis*-retinal oxime and *anti*-11-*cis*-retinal oxime, respectively). Spectra for retinyl esters and 11-*cis*-retinal oximes are shown in the lower panels. A gap was introduced in the chromatogram traces to remove a solvent change artifact occurring between the two isocratic elution steps. Color schemes are depicted in the legend. A and B are *RDH5^-/-^* cats 24-091 and 21-062, respectively. OD, oculus dexter; OS, oculus sinister.

***Figure S5. Injection site retinopathy.*** Red arrow denotes site of subretinal injection. Retinal damage is isolated to injection site (*RDH5^-/-^* cat 20-053).

***Table S1. Injected Animal Information.*** Identifying numbers, sex, status, and injection information of injected animals. OD; right eye, OS; left eye, *AC; area centralis*.

| **Identifying #** | **Status** | **Eye Treated** | **Sex** | **Age at injection (months)** | **Last Study Timepoint (months)** | **Transgene/Bleb** | **Total Bleb Surface Area (mm^2^)** | **Volume (μL)** | **Bleb encompassed AC (Y/N)** | **Developed AC degeneration (Y/N)** |
| --- | --- | --- | --- | --- | --- | --- | --- | --- | --- | --- |
| 18-142 | *RDH5^-/-^* | OD | F | 41 | 6 | RDH5 Large | 151 | ~440 | N | N |
| 20-055 | *RDH5^-/-^* | OS | M | 25 | 12 | RDH5 Large | 143 | ~200 | Y | N |
| 24-014 | *RDH5^-/-^* | OD | F | 6 | 12 | RDH5 Large | 90 | ~70 | Y | N |
| 24-015 | *RDH5^-/-^* | OD | M | 6 | 12 | RDH5 Large | 38 | ~75 | N | N |
| 19-117 | *RDH5^-/-^* | OD | F | 36 | 12 | RDH5 Large | 106 | ~180 | Y | N |
| 20-056 | *RDH5^-/-^* | OD | M | 12 | 12 | RDH5 Large | 173 | ~180 | N | N |
| 20-057 | *RDH5^-/-^* | OD | M | 25 | 12 | RDH5 Large | 90 | ~90 | Y | N |
| 23-038 | *RDH5^-/-^* | OD | F | 9 | 10 | RDH5 Large | 150 | ~400 | Y | N |
| 20-054 | *RDH5^-/-^* | OD | F | 17 | 48 | RDH5 Small | 22 | ~40 | Y | Y |
| 20-053 | *RDH5^-/-^* | OD | F | 17 | 48 | RDH5 Small | 28 | ~50 | Y | Y |
| 21-062 | *RDH5^-/-^* | OD/OS | F | 50 | 5 | RDH5 Large | 102/111 | ~130/100* | Y/N* | N |
| 24-091 | *RDH5^-/-^* | OD/OS | F | 12 | 5 | RDH5 Large | 123/36 | ~140/130* | Y/Y* | N |
| 18-046 | *RDH5^-/-^* | OD | M | 19 | 12 | RDH5^$^ | 194 | ~330 | N/A | N/A |
| 16-064 | *RDH5^-/-^* | OS | F | 44 | 7 | RDH5^$^ | 48 | ~150 | N/A | N/A |
| 15-024 | Control | OD/OS | M | 75 | 1 | GFP | 15/90 | ~50/150* | N/A | N/A |

*Indicates more than one eye was injected (OD/OS)
^$^AAV2 vector originally tested without triple capsid mutations

| **Identifying #** | **ERG** | **IHC** | **OCT** | **Semi-thin** | **Retinoid Analysis** | **Western** |
| --- | --- | --- | --- | --- | --- | --- |
| 18-142 | X | X | X |  |  |  |
| 20-055 | X |  | X |  |  |  |
| 24-014 | X | X | X |  |  |  |
| 24-015 | X | X | X |  |  |  |
| 19-117 | X |  | X |  |  |  |
| 20-056 | X |  | X |  |  |  |
| 20-057 | X | X | X |  |  |  |
| 23-038 | X | X | X |  |  |  |
| 20-054 |  |  | X | X |  |  |
| 20-053 |  |  | X | X |  |  |
| 21-062 |  |  | X |  | X |  |
| 24-091 |  | X | X |  | X | X |
| 18-046 | X | X | X |  |  |  |
| 16-064 | X | X | X |  |  |  |
| 15-024 |  | X | X |  |  |  |

***Table S2. Injected Animal Procedures and Analyses***. Identifying number and the corresponding procedures and analyses performed on injected animals.

***Table S3. Retinoid Analyses of RDH5^-/-^ and RDH5^+/+^ cats.*** Comparison of retinoid production in response to gene therapy treatment. A and B are *RDH5^-/-^* cats 24-091 and 21-062, respectively. WT: wildtype; OD: Right eye; OS: Left eye; RAL: retinaldehyde; RE: retinyl esters; AUC: area under the curve.

| **No.** | **Animal** | **Sex** | **Age at treatment (yrs.)** | **Eye/Section** | **Treated** | ***syn*-11-*cis*-RAL oxime**  **(AUC)** | **13-*cis*-RE**  **(AUC)** | **all-*trans*-RE**  **(AUC)** | **11-*cis*-RAL**  **(pmol)** | **13-*cis*-RE**  **(pmol)** | **all-*trans*-RE**  **(pmol)** | **13-*cis*-RE/all-*trans*-RE** | **11-*cis*-RAL/all-*trans*-RE** |
| --- | --- | --- | --- | --- | --- | --- | --- | --- | --- | --- | --- | --- | --- |
| 1 | A | Female | 1 | OD | Yes | 100.50 | 4.50 | 12.40 | 109.24 | 2.69 | 6.97 | 0.39 | 15.68 |
| 2 |  |  |  | OD | No | 74.30 | 57.67 | 29.50 | 80.76 | 34.53 | 16.57 | 2.08 | 4.87 |
| 3 | B | Female | 4.5 | OD | Yes | 110.00 | 2.80 | 13.40 | 119.57 | 1.68 | 7.53 | 0.22 | 15.88 |
| 4 |  |  |  | OD | No | 13.90 | 8.30 | 2.50 | 15.11 | 4.97 | 1.40 | 3.54 | 10.76 |
| 5 |  |  |  | OS | Yes | 85.30 | 2.40 | 6.00 | 92.72 | 1.44 | 3.37 | 0.43 | 27.51 |
| 6 |  |  |  | OS | No | 73.20 | 32.40 | 15.50 | 79.57 | 19.40 | 8.71 | 2.23 | 9.14 |
| 7 | C | WT Female | N/A | OD lateral | No | 113.00 | 1.40 | 6.20 | 122.83 | 0.84 | 3.48 | 0.24 | 35.26 |
| 8 |  |  |  | OS lateral | No | 79.40 | 1.10 | 4.20 | 86.30 | 0.66 | 2.36 | 0.28 | 36.58 |
| 9 |  |  |  | OS medial | No | 78.00 | 0.77 | 5.60 | 84.78 | 0.46 | 3.15 | 0.15 | 26.95 |

***Table S4. Dark-Adapted Scotopic ERG values.*** Comparison of b-wave (Naka Rushton) and a-wave (Birch and Hood) modeling parameters between treated and untreated eyes for dark-adapted scotopic ERGs. Normally distributed data was analyzed using a paired t-test and effect size was calculated using Hedge’s g. Not normally distributed data was analyzed using a paired Wilcoxon signed rank test (pseudomedian reported in mean difference column) and effect size was calculated using a matched-pairs rank-biserial correlation. Green highlight indicates significant p-value. PI; post-injection, CI; confidence interval, WT; wildtype.

| **Time PI** | **Parameter** | **Treated** | | **Untreated** | | **Mean Difference** | **Effect Size** | **p-value** | ***RDH5^-/-^* n** | **WT n** |
| --- | --- | --- | --- | --- | --- | --- | --- | --- | --- | --- |
|  |  | **Mean** | **SD** | **Mean** | **SD** |  |  |  |  |  |
| **3-months** | *Td** | 1.43 | 0.26 | 1.38 | 0.29 | 0.01, 95% CI [-0.04,0.28] | 0.24, 95% CI [-0.57,0.81] | 0.69 | 6 | 9 |
|  | *LogS* | 2.16 | 0.16 | 2.19 | 0.16 | -0.29, 95% CI [-0.08,0.02] | -0.58, 95% CI [-1.31,0.20] | 0.10 |  |  |
|  | *LogRmax* | 2.26 | 0.10 | 2.21 | 0.11 | 0.05, 95% CI [-0.05,0.15] | 0.42, 95% CI [-0.32,1.12] | 0.27 |  |  |
|  | *Vm* | 363.42 | 66.53 | 323.42 | 88.75 | 40, 95% CI  [- 46.05,126.1] | 0.41, 95% CI [-0.32,1.11] | 0.29 |  |  |
|  | *N* | 1.04 | 0.15 | 1.00 | 0.09 | 0.04, 95% CI [-0.10,0.18] | 0.23, 95% CI [-0.47,0.91] | 0.53 |  |  |
|  | *Logk* | -2.52 | 0.31 | -2.58 | 0.26 | 0.06, 95% CI [-0.05,0.18] | 0.47, 95% CI [-0.28,1.19] | 0.23 |  |  |
| **6-months** | *Td* | 1.43 | 0.23 | 1.41 | 0.27 | 0.02, 95% CI [-0.09,0.12] | 0.15, 95% CI [-0.51,0.79] | 0.69 | 7 |  |
|  | *LogS* | 2.15 | 0.22 | 2.12 | 0.20 | 0.03, 95% CI [-0.07,0.13] | 0.22, 95% CI [-0.44,0.87] | 0.52 |  |  |
|  | *LogRmax* | 2.23 | 0.15 | 2.13 | 0.17 | 0.10, 95% CI [-0.01,0.21] | 0.71, 95% CI [-0.07,1.44] | 0.08 |  |  |
|  | *Vm* | 339.36 | 147.56 | 287.86 | 185.24 | 51.5, 95% CI [-16.83,119.83] | 0.61, 95% CI [-0.14,1.31] | 0.11 |  |  |
|  | *N** | 1.03 | 0.11 | 0.97 | 0.06 | 0.05, 95% CI [0.01,0.11] | 1, 95% CI [1.00,1.00] | 0.02 |  |  |
|  | *Logk* | -2.44 | 0.25 | -2.42 | 0.28 | -0.02, 95% CI [-0.07,0.04] | -0.22, 95% CI [-0.87,0.44] | 0.52 |  |  |
| **1-year** | *Td* | 1.57 | 0.25 | 1.42 | 0.13 | 0.14, 95% CI [-0.31,0.59] | 0.44, 95% CI [-0.34,1.17] | 0.31 | 3 |  |
|  | *LogS* | 2.11 | 0.05 | 2.15 | 0.17 | -0.04, 95% CI [-0.40,0.32] | -0.17, 95% CI [-0.80,0.51] | 0.66 |  |  |
|  | *LogRmax* | 2.20 | 0.21 | 2.09 | 0.22 | 0.12, 95% CI [-0.05,0.28] | 0.99, 95% CI [-0.12,2.07] | 0.09 |  |  |
|  | *Vm* | 347.00 | 213.94 | 287.67 | 219.40 | 59.33, 95% CI [17.96,100.7] | 2.01, 95% CI [0.15,3.95] | 0.03 |  |  |
|  | *N* | 0.96 | 0.04 | 1.00 | 0.03 | -0.04, 95% CI [-0.08,0.01] | -1.27, 95% CI [-2.58,0.04] | 0.06 |  |  |
|  | *Logk* | -2.77 | 0.32 | -2.76 | 0.32 | -0.01, 95% CI [-0.22,0.21] | -0.03, 95% CI [-0.66,0.62] | 0.95 |  |  |

*Indicates data was not normally distributed and a Wilcoxon signed rank test was performed. All other data was analyzed using a paired t-test.

***Table S5. Photopic ERG Values.*** Comparison of a-wave, b-wave, and flicker amplitudes and peak times between treated and untreated eyes using standard photopic ERG luminances. Green highlight indicates significant p-value. Normally distributed data was analyzed using a paired t-test and effect size was calculated using Hedge’s g. Not normally distributed data was analyzed using a paired Wilcoxon signed rank test (pseudomedian reported in mean difference column) and effect size was calculated using a matched-pairs rank-biserial correlation. PI; post-injection, CI; confidence interval, WT; wildtype.

| **Time PI** | **Parameter** | **Flash  ( cd⋅s/m^2^)** | **Treated** | | **Untreated** | | **Mean Difference** | **Effect Size** | **p-value** | ***RDH5^-/-^* n** | **WT n** |
| --- | --- | --- | --- | --- | --- | --- | --- | --- | --- | --- | --- |
|  |  |  | Mean | SD | Mean | SD |  |  |  |  |  |
| **3-months** | A-Wave Amp | 2.482 | 4.23 | 2.11 | 4.25 | 2.78 | -0.02, 95% CI [-1.29,1.24] | -0.02, 95% CI [-0.69,0.66] | 0.96 | 6 | 8 |
|  |  | 7.2 | 6.91 | 1.78 | 5.69 | 2.28 | 1.22, 95% CI [-1.28,3.73] | 0.43, 95% CI [-0.31,1.13] | 0.27 |  |  |
|  |  | 23 | 10.29 | 2.51 | 7.90 | 2.29 | 2.39, 95% CI [-0.35,5.13] | 0.77, 95% CI [-0.07,1.56] | 0.08 |  |  |
|  | A-Wave Time | 2.482 | 10.17 | 1.60 | 10.58 | 1.43 | -0.42, 95% CI [-2.53,1.69] | -0.17, 95% CI [-0.85,0.51] | 0.63 |  |  |
|  |  | 7.2* | 9.75 | 1.04 | 9.92 | 1.36 | -0.25, 90% CI [-0.75,0.50] | -0.24, 95% CI [-0.81,0.57] | 0.67 |  |  |
|  |  | 23 | 8.67 | 1.17 | 8.25 | 0.61 | 0.42, 95% CI [-0.93,1.76] | 0.27, 95% CI [-0.43,0.95] | 0.46 |  |  |
|  | B-Wave Amp | 2.482 | 28.69 | 9.63 | 24.79 | 12.61 | 3.90, 95% CI [-2.07,9.88] | 0.58, 95% CI [-0.20,1.31] | 0.15 |  |  |
|  |  | 7.2 | 37.73 | 11.91 | 35.69 | 16.72 | 2.03, 95% CI [-8.00,12.07] | 0.18, 95% CI [-0.51,0.85] | 0.62 |  |  |
|  |  | 23 | 33.60 | 10.22 | 32.02 | 14.00 | 1.59, 95% CI [-4.34,7.52] | 0.24, 95% CI [-0.46,0.91] | 0.52 |  |  |
|  | B-Wave Time | 2.482* | 24.08 | 1.02 | 23.33 | 1.37 | 0.85, 60% CI [0.50,1.50] | 1, 95% CI [1.00,1.00] | 0.10 |  |  |
|  |  | 7.2* | 25.92 | 1.28 | 25.33 | 1.47 | 1.75, CI not estimable$ | 1, 95% CI [1.00,1.00] | 0.37 |  |  |
|  |  | 23* | 25.75 | 2.16 | 25.92 | 2.04 | -0.25, 80% CI [-0.75,0.50,] | -0.29, 95% CI [-0.83,0.53] | 0.59 |  |  |
|  | Flicker Amp | 3 (33 Hz) | 24.64 | 12.27 | 20.47 | 9.58 | 4.17, 95% CI [-1.30,9.65] | 0.67, 95% CI [-0.14,1.43] | 0.11 |  |  |
|  | Flicker Time | 3 (33 Hz) | 11.92 | 1.07 | 12.08 | 2.13 | -0.17, 95% CI [-3.20,2.87] | -0.05, 95% CI [-0.72,0.63] | 0.89 |  |  |
| **6-months** | A-Wave Amp | 2.482 | 4.24 | 1.34 | 3.18 | 0.93 | 1.06, 95% CI [-0.43,2.56] | 0.57, 95% CI [-0.16,1.27] | 0.13 | 7 |  |
|  |  | 7.2 | 5.65 | 2.02 | 4.85 | 1.62 | 0.80, 95% CI [-0.93,2.53] | 0.37, 95% CI [-0.32,1.03] | 0.30 |  |  |
|  |  | 23 | 8.26 | 1.96 | 7.65 | 2.84 | 0.61, 95% CI [-1.05,2.28] | 0.30, 95% CI [-0.38,0.95] | 0.40 |  |  |
|  | A-Wave Time | 2.482 | 10.29 | 1.32 | 11.14 | 1.89 | -0.86, 95% CI [-3.04,1.32] | -0.32, 95% CI [-0.97,0.36] | 0.37 |  |  |
|  |  | 7.2 | 8.86 | 1.25 | 9.21 | 1.44 | -0.36, 95% CI [-2.12,1.41] | -0.16, 95% CI [-0.81,0.49] | 0.64 |  |  |
|  |  | 23 | 8.57 | 1.64 | 9.50 | 1.41 | -0.93, 95% CI [-1.61,-0.25] | -1.10, 95% CI [-1.97,-0.19] | 0.02 |  |  |
|  | B-Wave Amp | 2.482 | 23.38 | 9.49 | 19.92 | 14.50 | 3.46, 95% CI [-3.19,10.11] | 0.42, 95% CI [-0.28,1.09] | 0.25 |  |  |
|  |  | 7.2 | 30.10 | 12.10 | 29.25 | 20.88 | 0.85, 95% CI [-9.14,10.85] | 0.07, 95% CI [-0.58,0.71] | 0.84 |  |  |
|  |  | 23 | 27.86 | 9.43 | 26.88 | 15.90 | 0.97, 95% CI [-7.72,9.67] | 0.09, 95% CI [-0.56,0.73] | 0.79 |  |  |
|  | B-Wave Time | 2.482 | 25.36 | 2.12 | 24.43 | 2.95 | 0.93, 95% CI [-0.44,2.30] | 0.54, 95% CI [-0.18,1.24] | 0.15 |  |  |
|  |  | 7.2 | 27.07 | 1.99 | 26.71 | 2.21 | 0.36, 95% CI [-0.16,0.87] | 0.56, 95% CI [-0.17,1.25] | 0.14 |  |  |
|  |  | 23* | 26.43 | 1.37 | 27.36 | 2.34 | -0.5, 80% CI [-3.25,1.27e-05] | -0.60, 95% CI [-0.91,0.13] | 0.34 |  |  |
|  | Flicker Amp | 3 (33 Hz) | 24.61 | 10.87 | 21.51 | 14.82 | 3.10, 95% CI [-3.37,9.58] | 0.38, 95% CI [-0.31,1.05] | 0.29 |  |  |
|  | Flicker Time | 3 (33 Hz) | 13.36 | 1.00 | 12.96 | 1.42 | 0.4, 95% CI [-0.90,1.70] | 0.25, 95% CI [-0.42,0.90] | 0.48 |  |  |
| **1-year** | A-Wave Amp | 2.482 | 3.36 | 1.71 | 2.51 | 0.68 | 0.84, 95% CI [-2.47,4.15] | 0.36, 95% CI [-0.39,1.04] | 0.39 | 3 |  |
|  |  | 7.2 | 7.33 | 2.95 | 5.04 | 1.77 | 2.28, 95% CI [-2.79,7.36] | 0.63, 95% CI [-0.26,1.45] | 0.19 |  |  |
|  |  | 23 | 7.83 | 3.30 | 6.40 | 1.99 | 1.43, 95% CI [-1.84,4.70] | 0.61, 95% CI [-0.27,1.43] | 0.20 |  |  |
|  | A-Wave Time | 2.482 | 10 | 0.87 | 9.83 | 0.76 | 0.17, 95% CI [-3.63,3.96] | 0.06, 95% CI [-0.59,0.70] | 0.87 |  |  |
|  |  | 7.2 | 8.17 | 1.26 | 10.17 | 1.76 | -2.0, 95% CI [-8.57,4.57] | -0.43, 95% CI [-1.14,0.35] | 0.32 |  |  |
|  |  | 23* | 6.67 | 0.76 | 7.17 | 1.26 | -0.37, CI not estimable$ | -0.67, 95% CI [-0.97,0.39] | 0.41 |  |  |
|  | B-Wave Amp | 2.482 | 29.88 | 16.87 | 18.78 | 7.85 | 11.10, 95% CI [-13.86,36.07] | 0.62, 95% CI [-0.26,1.44] | 0.20 |  |  |
|  |  | 7.2 | 35.40 | 20.58 | 28.99 | 14.37 | 6.41, 95% CI [-20.67,33.48] | -0.43, 95% CI [-1.14,0.35] | 0.42 |  |  |
|  |  | 23 | 33.35 | 20.30 | 25.16 | 13.18 | 8.19, 95% CI [-10.85,27.22] | 0.60, 95% CI [-0.27,1.41] | 0.21 |  |  |
|  | B-Wave Time | 2.482 | 23.33 | 2.02 | 22.67 | 1.26 | 0.67, 95% CI [-1.23,2.57] | 0.49, 95% CI [-0.32,1.24] | 0.27 |  |  |
|  |  | 7.2* | 25.33 | 2.25 | 24.67 | 1.76 | 1.00, CI not estimable$ | 1.00, 95% CI [1.00,1.00] | 0.35 |  |  |
|  |  | 23* | 26 | 3 | 25.17 | 3.33 | 2.5, CI not estimable$ | 1.00, 95% CI [1.00,1.00] | 1.00 |  |  |
|  | Flicker Amp | 3 (33 Hz) | 28.50 | 18.43 | 19.38 | 10.81 | 9.11, 95% CI [-12.94,31.17] | 0.58, 95% CI [-0.28,1.37] | 0.22 |  |  |
|  | Flicker Time | 3 (33 Hz)* | 13 | 1.00 | 11 | 1.00 | 3.00, CI not estimable$ | 1.00, 95% CI [1.00,1.00] | 0.35 |  |  |

*Indicates data was not normally distributed and a Wilcoxon signed rank test was performed.

$Confidence interval not estimable due to low sample size, zero differences, and tied ranks in the paired differences

***Table S6. Rod Recovery ERG Values.*** Comparison of percent rod recovery following light exposure between treated and untreated eyes. Green highlight indicates significant p-value. Normally distributed data was analyzed using a paired t-test and effect size was calculated using Hedge’s g. Not normally distributed data was analyzed using a paired Wilcoxon signed rank test (pseudomedian reported in mean difference column) and effect size was calculated using a matched-pairs rank-biserial correlation. PI; post-injection, CI; confidence interval, WT; wildtype.

| **Time PI** | **Treated** | | **Untreated** | | **Mean Difference** | **Effect Size** | **p-value** | ***RDH5^-/-^* n** | **WT n** |
| --- | --- | --- | --- | --- | --- | --- | --- | --- | --- |
|  | **Mean** | **SD** | **Mean** | **SD** |  |  |  |  |  |
| **3-month** | 33.11 | 13.63 | 14.54 | 12.55 | 18.57, 95% CI [11.82,25.33] | 2.21, 95% CI [0.82,3.57] | <0.001 | 7 | 6 |
| **6-month*** | 37.61 | 19.51 | 13.66 | 10.28 | 21.86, 95% CI [14.78,37.76] | 1, 95% CI [1.00,1.00] | 0.008 | 8 |  |
| **1-year** | 21.05 | 9.36 | 9.41 | 10.67 | 11.64, 95% CI [1.86,21.41] | 1.18, 95% CI [0.10,2.21] | 0.03 | 5 |  |

*Indicates data was not normally distributed and a Wilcoxon signed rank test was performed.

***Table S7. Bright Flash and Cone Recovery ERG Values.*** Comparison of percent bright flash baseline for BF a-wave % of BL at step 25, BF b-wave % of BL at step 25, CR a-wave % of BF BL at 120 minutes, and CR b-wave % of BF BL at 120 minutes between treated and untreated eyes. Green highlight indicates significant p-value. Normally distributed data was analyzed using a paired t-test and effect size was calculated using Hedge’s g. Not normally distributed data was analyzed using a paired Wilcoxon signed rank test (pseudomedian reported in mean difference column) and effect size was calculated using a matched-pairs rank-biserial correlation. BL; baseline, PI; post-injection, BF; bright flash, CR; cone recovery, CI; confidence interval, WT; wildtype.

| **Time PI** | **Parameter (% of BF BL)** | **Treated** | | **Untreated** | | **Mean Difference** | **Effect Size** | **p-value** | ***RDH5^-/-^* n** | **WT n** |
| --- | --- | --- | --- | --- | --- | --- | --- | --- | --- | --- |
|  |  | **Mean** | **SD** | **Mean** | **SD** |  |  |  |  |  |
| **3-months** | BF A-Wave | 59.33 | 14.93 | 62.63 | 15.17 | -3.33, 95% CI [-26.69,20.08] | -0.12, 95% CI [-0.80,0.56] | 0.73 | 6 | 8 |
|  | BF B-Wave | 51.22 | 9.05 | 40.01 | 15.67 | 11.22, 95% CI [-2.41,24.84] | 0.73, 95% CI [-0.10,1.51] | 0.09 |  |  |
|  | CR A-Wave | 61.03 | 24.21 | 42.5 | 14.88 | 18.53, 95% CI [-12.29,49.35] | 0.53, 95% CI [-0.23,1.25] | 0.18 |  |  |
|  | CR B-Wave | 56.40 | 20.05 | 41.28 | 24.25 | 15.12, 95% CI [6.22,24.02] | 1.50, 95% CI [0.35,2.60] | 0.007 |  |  |
| **6-months** | BF A-Wave* | 65.04 | 13.94 | 66.01 | 26.51 | 4.46, 95% CI [-20.89,12.72] | 0.29, 95% CI [-0.49,0.81] | 0.58 | 7 |  |
|  | BF B-Wave* | 52.44 | 13.78 | 46.44 | 13.92 | 10.31, 95% CI [-8.89,15.65] | 0.50, 95% CI [-0.27,0.88] | 0.30 |  |  |
|  | CR A-Wave | 62.74 | 9.54 | 55.33 | 16.77 | 7.41, 95% CI [-13.54,28.37] | 0.28, 95% CI [-0.39,0.94] | 0.42 |  |  |
|  | CR B-Wave | 52.05 | 12.02 | 35.48 | 6.80 | 16.57, 95% CI [6.89,26.25] | 1.38, 95% CI [0.36,2.35] | 0.006 |  |  |
| **1-year** | BF A-Wave* | 65.96 | 4.02 | 60.08 | 26.67 | 8.67, 75% CI [-21.22,21.81] | 0.33, 95% CI [,-0.70,0.92] | 0.75 | 3 |  |
|  | BF B-Wave* | 51.30 | 5.05 | 36.26 | 9.46 | 14.47, 75% CI [7.21,25.16] | 1, 95% CI [1.00,1.00] | 0.25 |  |  |
|  | CR A-Wave | 57.08 | 19.11 | 46.93 | 6.40 | 10.15, 95% CI [-42.15,62.46] | 0.27, 95% CI [-0.44,0.93] | 0.49 |  |  |
|  | CR B-Wave | 53.32 | 12.41 | 29.62 | 5.62 | 23.71, 95% CI [5.43,41.99] | 1.82, 95% CI [0.11,3.59] | 0.03 |  |  |

*Indicates data was not normally distributed and a Wilcoxon signed rank test was performed.
